# Utilizing symbiotic relationships and assisted migration in restoration to cope with multiple stressors, and the legacy of invasive species

**DOI:** 10.3389/frmbi.2024.1331341

**Published:** 2024-03-26

**Authors:** Lisa M. Markovchick, Abril Belgara-Andrew, Duncan Richard, Tessa Deringer, Kevin C. Grady, Kevin R. Hultine, Gerard J. Allan, Thomas G. Whitham, José Ignacio Querejeta, Catherine A. Gehring

**Affiliations:** ^1^ Department of Biological Sciences, Northern Arizona University, Flagstaff, AZ, United States; ^2^ Center for Adaptable Western Landscapes, Northern Arizona University, Flagstaff, AZ, United States; ^3^ Rocky Mountain Research Station, United States Department of Agriculture (USDA) Forest Service, Flagstaff, AZ, United States; ^4^ School of Forestry, Northern Arizona University, Flagstaff, AZ, United States; ^5^ Department of Research, Conservation and Collections, Desert Botanical Garden, Phoenix, AZ, United States; ^6^ Soil and Water Conservation Group, Spanish National Research Council, Centro de Edafología y Biología Aplicada del Segura (CEBAS)-Consejo Superior de Investigaciones Científicas (CSIC), Murcia, Spain

**Keywords:** assisted migration, mycorrhiza, restoration, ecotype, invasive species, physiology, *Populus fremontii*, translocation

## Abstract

**Introduction:**

Climate change has increased the need for forest restoration, but low planting success and limited availability of planting materials hamper these efforts. Invasive plants and their soil legacies can further reduce restoration success. Thus, strategies that optimize restoration are crucial. Assisted migration and inoculation with native microbial symbiont communities have great potential to increase restoration success. However, assisted migrants can still show reduced survival compared to local provenances depending on transfer distance. Inoculation with mycorrhizal fungi, effective if well-matched to plants and site conditions, can have neutral to negative results with poor pairings. Few studies have examined the interaction between these two strategies in realistic field environments where native plants experience the combined effects of soil legacies left by invasive plants and the drought conditions that result from a warming, drying climate.

**Methods:**

We planted two ecotypes (local climate and warmer climate) of *Populus fremontii* (Fremont cottonwoods), in soils with and without legacies of invasion by *Tamarix* spp. (tamarisk), and with and without addition of native mycorrhizal fungi and other soil biota from the warmer climate.

**Results:**

Four main results emerged. 1) First year survival in soil legacies left behind after tamarisk invasion and removal was less than one tenth of survival in soil without a tamarisk legacy. 2) Actively restoring soil communities after tamarisk removal tripled first year cottonwood survival for both ecotypes, but only improved survival of the warmer, assisted migrant ecotype trees in year two. 3) Actively restoring soil communities in areas without a tamarisk history reduced first year survival for both ecotypes, but improved survival of the warmer, assisted migrant ecotype trees in year two. 4) By the second year, inoculated assisted migrants survived at five times the rate of inoculated trees from the local ecotype.

**Discussion:**

Results emphasize the detrimental effects of soil legacies left after tamarisk invasion and removal, the efficacy of assisted migration and restoring soil communities alongside plants, and the need to thoughtfully optimize pairings between plants, fungi, and site conditions.

## 1 Introduction

Climate change is increasing the need for, and importance of, natural regeneration, replanting, and restoration. In addition to the importance of sequestering carbon, replanting and restoration is increasingly needed following natural disasters that have increased in frequency, size and severity (National Academies of Sciences, Engineering, and Medicine, 2020; Parks and Abatzoglou, 2020; Fargione et al., 2021; Parks et al., 2023). However, planting material shortages, and climate change constrain both restoration efforts and their success (Wheeler et al., 2015; National Academies of Sciences, Engineering, and Medicine, 2020; Fargione et al., 2021; Simonson et al., 2021). For these reasons, optimizing the effectiveness of restoration is becoming increasingly important.

Climate change may outpace the ability of plants to adapt or migrate via natural dispersal, such that the practice of using local seed stock and planting material for restoration may not continue to be optimal (Whitham et al., 2020). Introducing additional genetic diversity into ecosystems using strategic assisted migration of plant provenances (local populations), or species, from warmer or drier locations, has been suggested as a strategy to help ecosystems adapt (Etterson et al., 2020; Gomory et al., 2020; Saenz-Romero et al., 2021). However, assisted migrant plants can be more susceptible to disease and can yield lower survival than local provenances due to adaptations to local temperature regimes including cold extremes (Tiscar et al., 2018; Cooper et al., 2019; Simler et al., 2019). Michalet et al. (2023) also warn of consequences for fire risk and climate buffering. Translocated Fremont cottonwood demonstrate survival correlated with transfer distance or temperature difference. Translocated Fremont cottonwoods (*Populus fremontii*) can have survival comparable to local provenances if the temperature differences between transplanted and home site are small, but survival reduced by up to 30% in trees transferred across greater temperature differences, especially those upwards of 3°C (Grady et al., 2011; 2015; Cooper et al., 2019). The local suitability of Fremont cottonwood for the shifting climate is affected by adaptations of their population and ecotype (Ikeda et al., 2017; Cooper et al., 2019; Blasini et al., 2021; 2022). Trees from the warmer Sonoran Desert (SD) ecotype have developed physiological traits which promote water movement through the plant for evaporative cooling (but may leave trees susceptible to drought-driven stress or frost), while trees from the cooler Mogollon Rim (MR) ecotype have developed traits which reduce their susceptibility to frost (which may help with drought-resilience, but result in more susceptibility to heat stress; Hultine et al., 2020; Blasini et al., 2021; 2022). Consequently, regional and population level adaptations can be an important consideration for sourcing planting material, but it is not always clear how to optimize provenance selection. There is also an urgent need for large-scale, multi-species experimental studies to provide evidence that assisted migration is advisable given local adaptation of plants to other aspects of the abiotic environment besides temperature, and to address implications for interspecies interactions including those between plants and soil microbes (Bucharova, 2017).

The need for experimental studies that assess the interactions between the assisted migration of plants and the microbiome intersects with another strategy suggested to improve restoration outcomes, stretch the impact of available planting material, and help assisted migrant plants adapt to new sites. Active restoration of diverse native mycorrhizal fungal communities symbiotic with native plant roots has shown great potential as a tool to increase revegetation success. Many kinds of disturbances negatively impact mycorrhizal fungi, such as pollution deposition, land use changes, invasion by exotic species, pesticide application, and climate change (e.g. Egerton-Warburton and Allen, 2000; Meinhardt and Gehring, 2012; Helander et al., 2018; León-Sánchez et al., 2018; Querejeta et al., 2021; IPBES, 2023). In particular, invasion by exotic plant species has been documented to simultaneously reduce native plant biomass, survival, and diversity in conjunction with reducing or shifting their mycorrhizal fungal partner communities. Invasive plants including *Cynodon dactylon* (Bermuda grass), *Calamagrostis epigejos* (bush grass), *Avena barbata* (slender wild oats), *Bromus hordeaceus* (soft chess), *Bothriochloa bladhii* (old world bluestem), *Tamarix* spp. (tamarisk), garlic mustard (*Alliaria petiolata*) and others (Hawkes et al., 2006; Wolfe et al., 2008; Wilson et al., 2012; Endresz and Kalapos, 2013) have all been shown to alter mycorrhizal communities and impact native plant productivity and survival.

In the native riparian ecosystems of the southwestern United States, invasive tamarisk (*Tamrix* spp.) trees have repeatedly been shown to negatively impact the survival and biomass of Fremont cottonwood trees (*Populus fremontii*), and concurrently deplete and shift their active mycorrhizal fungal communities (e.g. Beauchamp et al., 2005; 2006; Gitlin et al., 2006; Meinhardt and Gehring, 2012; Hultine et al., 2015). Decreases in diversity and shifts in mycorrhizal fungal communities from invasive species seem to arise from two potential but not mutually exclusive causes, changes in soil chemistry caused by the invasive species, and reductions and shifts in the microbial and/or mycorrhizal fungal community due to reduced native plant hosts. For example, *Solidago canadensis* (Canada goldenrod) can shift the fungal community to one that promotes its own competitiveness over that of native plants (Zhang et al., 2010), and some invasive plant species such as tamarisk and garlic mustard are known to alter soil salinity, nitrate concentrations, or produce antifungal phytochemicals and allelochemicals (Lesica and DeLuca, 2004; Wolfe et al., 2008; Meinhardt and Gehring, 2012). However, it remains unclear if changes to soil chemistry and/or the impacts on mycorrhizal fungi and cottonwood survival and biomass persist as a legacy in the soil (soil legacy) once invasive tamarisk plants are removed, or the degree to which active additions of soil microbial communities could offset these effects.

Actively adding diverse, native soil microbial communities appropriate to plant host species, and provenances (often done via actively collecting, cultivating, and adding live soil inoculum from reference or non-degraded sites into degraded sites along with planting) following invasive plant removal could improve restoration outcomes. In particular, we use this technique to restore native mycorrhizal fungal communities, due to the body of literature demonstrating its success with mycorrhizal fungi (referred to hereafter as mycorrhizal restoration), and the research on Fremont cottonwoods showing that tamarisk negatively affects cottonwood survival, biomass, and mycorrhizal associations. Restoring mycorrhizal fungal communities in this way has been demonstrated to promote survival, growth, and diversity of plant communities, if thoughtfully incorporated into restoration planning in conjunction with plant provenances and communities, planting palettes and site conditions (e.g. Rua et al., 2016; Wubs et al., 2016; Koziol and Bever, 2017). At least one meta-analysis has shown that the benefits improve with time since planting (Neuenkamp et al., 2019). These findings concur with broader science reflecting that the ability of organisms to survive and adapt is likely dependent on the confluence of their own traits and adaptations with those of their microbiome (Zilber-Rosenberg and Rosenberg, 2008; Bordenstein and Theis, 2015 ; Whitham et al., 2020). In fact, because of shorter life cycles, microbiota like mycorrhizal fungi may be particularly crucial to promoting swift adaptation to climate change and rehabilitation of degraded systems (Wilkinson and Dickinson, 1995; Coban et al., 2022; Allsup et al., 2023; Argüelles-Moyao and Galicia, 2023).

However, consideration of factors including timing, soil properties, plant species and plant provenance have proven integral in achieving optimal results when implementing mycorrhizal restoration (e.g., Johnson et al., 1992; Mortimer et al., 2005; Maltz and Treseder, 2015). Neutral to negative effects occur when these factors are not adequately addressed or mass-produced products are utilized (e.g., Maltz and Treseder, 2015; Rua et al., 2016; Saloman et al., 2022), or when plant roots do not gain direct contact with active mycorrhizal fungi that are provided by mycorrhizal symbioses living on adjacent native plants (Jones et al., 2003; Grünfeld et al., 2022). Thus, it remains unclear how well optimized plant and fungal pairings must be, and how assisted migration and the tendency for co-adaptation between local soil, mycorrhizal fungi, and plants indicated by other studies (e.g. Johnson et al., 1992; 2010; 2014; Rua et al., 2016) will interact. Filling these knowledge gaps can inform the cost/benefit calculations for implementing active restoration of native mycorrhizal communities when it is needed (for example, see Hart et al., 2017), and assist in the design of inoculum and plant provenance combinations that best harness biotic co-adaptation and site conditions.

To address these knowledge gaps, we investigated the interactions between three experimental treatments under stressful drought conditions: assisted migration (from a warmer ecotype), inoculation with mycorrhizal fungi and associated soil organisms (collected from beneath a warmer ecotype), and soil legacies left by invasive tamarisk. We focus here on Fremont cottonwood due to its foundational nature in riparian communities of the arid southwest, extensive data on its ecotypes and ecophysiology, and the immense loss of riparian areas experienced in the United States. Riparian arteries of the southwestern U.S. comprise less than 0.5% and 2% of the land area in Arizona and the southwest, respectively, but support a disproportionate 60 to 75% of the wildlife (Poff et al., 2012). Best estimates placed the loss of these riparian habitats at 90% (Zaimes, 2007). Previous studies have also demonstrated that warmer, supraoptimal temperatures reduce canopy gas exchange via reduced stomatal conductance (Grady et al., 2013), increase plant stress (Hultine et al., 2013), and are expected to create predictable, linear losses in tree productivity (Grady et al., 2011). Importantly, with increasing aridity and record temperatures, recent heat waves have exceeded T_crit_, the temperature at which Photosystem II is disrupted in cottonwoods, requiring evaporative leaf cooling to maintain photosynthesis. Combined with declining stream flows and water tables, these high temperatures threaten some cottonwood with catastrophic die offs (Moran et al., 2023). Because mycorrhizal associations are known to assist some plants in water uptake, movement, and water use efficiency (e.g. Querejeta et al., 2003; 2006; 2007; Egerton-Warburton et al., 2007), they could play an important role in affecting the survival of these trees and in restoration. We used provenance trials with assisted migration to replant a riparian corridor and floodplain after tamarisk invasion. We planted trees from six provenances, three each from two contrasting Fremont cottonwood ecotypes (warmer SD and cooler MR) at a cooler MR ecotype site.

Given prior research regarding the negative impacts of tamarisk neighbors on native cottonwoods and their mycorrhizal communities (Meinhardt and Gehring, 2012), the long-term effects of mycorrhizal disruptions and dispersal challenges (Peay et al., 2010; Pankova et al., 2018; Anthony et al., 2019), benefits of mycorrhizal inoculation (Neuenkamp et al., 2019), and challenges with assisted migration (Grady et al., 2015; Tiscar et al., 2018; Cooper et al., 2019; Simler et al., 2019), we hypothesized that: 1) the soil legacy left by prior tamarisk invasion would reduce plant survival, even after tamarisk removal; mycorrhizal restoration would improve the survival of trees from both ecotypes 2) in areas with a tamarisk soil legacy and 3) also in areas without a tamarisk history (since even non-tamarisk areas were degraded and inoculum was native and regionally appropriate); 4) assisted migrant trees from the warmer SD ecotype would have survival rates equal to or lower than those for trees from the local MR ecotype, regardless of tamarisk and inoculation treatments (given prior results from assisted migration trials with Fremont cottonwoods; e.g. Grady et al., 2011, 2015; Cooper et al., 2019).

## 2 Materials and methods

### 2.1 Common garden planting site

We chose a site along the Little Colorado River near Cameron, AZ, to establish an experimental common garden (35.71923, −111.3194). The site consists of 22 hectares in a riparian corridor and flood plain. The site falls within the cooler Mogollon Rim ecotype (MR) of Fremont cottonwoods (*Populus fremontii*). The site had been used as source for gravel since at least 2007. All areas included within the site were degraded. Native shrubs and trees that could provide the direct contact with mycorrhizal roots needed for *in situ* mycorrhizal inoculation of plantings were absent from the experimental areas. Areas without tamarisk often contained other invasive species such as *Alhagi maurorum* (camelthorn).

### 2.2 Cottonwoods and planting methods for the field experiment

Cottonwood cuttings were collected from trees at least 20 m apart during the 2014–2015 winter. Cuttings were collected from six provenances across two ecotypes (Mogollon Rim, MR; Sonoran Desert, SD; Blasini et al., 2021, 2022). The soil and climate conditions at the common garden and tree source provenances (populations) are shown in **Table 1**, along with the number of trees used in the experiment from each source population. The three source provenances from the SD ecotype have mean annual temperatures (MAT) that are warmer than the common garden planting site (**Table 1**; ranging from 0.1° to 1.8°C warmer, as measured in 2018). The three source provenances from the MR ecotype have mean annual temperatures (MAT) that are the same as, or cooler than, the common garden planting site (**Table 1**; ranging from 0° difference to 4.9°C cooler, based on 30 year averages). Cottonwoods from the cooler, Mogollon Rim (MR) ecotype express traits associated with frost and drought protection (e.g., later seasonal leaf flush, smaller xylem vessels, reduced stomatal density; Blasini et al., 2021, 2022). Alternatively, cottonwoods from the warmer, Sonoran Desert (SD) ecotype have traits associated with heat tolerance (e.g. hydraulic efficiency and evaporative cooling; Blasini et al., 2021, 2022), which may reduce tolerance to droughts that are increasing in frequency and severity in the western United States (Moran et al., 2023). Cottonwood cuttings for all treatments were propagated and grown in a soil mixture consisting of peat moss, perlite, and vermiculite in 10.16 cm by 76.20 cm pots to encourage deep root systems with minimal soil microbes. Cuttings were grown in the greenhouse under normal day and night cycles for two years and watered every third day. A few cuttings were screened for the presence of ectomycorrhizas prior to planting and none were observed.

**Table 1:** Climate and soil characteristics for the common garden and plant and inoculum ecotype source locations.

Source Population, River (Code)	Keams Canyon, Little Colorado River (KKH-OPI)	Jack Rabbit, Little Colorado River (JLA-JAK)	Citadel Wash, Little Colorado River (CLF-LCR)	Little Colorado River Common Garden, Little Colorado River (LCR)	Kingman, Willow Creek (KWF-WIL)	Bullpen, Clear Creek (BCE-BUL)	Horseshoe Ranch, Agua Fria (CAF-AUG)
**Ecotype**	**MR**	**MR**	**MR**	**MR**	**SD**	**SD**	**SD**
**Latitude**	35.81152	34.96	35.6088	35.71923	35.143	34.53972	34.25671
**Longitude**	-110.16958	-110.436	−111.31369	-111.3194	-113.54284	-111.6966	-112.06617
**Elevation (m)**	1982	1510	1349	1331	1210	1254	1061
**Annual Precipitation (mm)**	213.8	194.3	184.17	174.34	246.52	426.74	408.56
**Min. Temperature (°C)**	2.1	4.2	7.2	7.2	7.2	7.9	10.6
**Max. Temperature (°C)**	18.8	22.4	23.3	23.4	24.2	24.3	25.4
**MAT (°C)**	10.4	13.3	15.3	15.3	15.7	16.1	18.0
**MAT Transfer (°C)**	4.9	2.0	0	–	-0.4	-0.8	-2.7
**Max VPD (hPa)**	19.4	25.62	27.77	27.87	28.06	27.76	29.18
**Soil Series**	Tewa very fine sandy loam	Ives Soils	Jocity-Joraibi-Navajo-Riverwash complex	Jocity-Joraibi-Navajo-Riverwash complex/Torrifluvents, saline	Arizo-Franconia/Riverwash complex	Anthony fine sandy loam	Barkerville Cobbly Sandy Loam
**Soil Content**	59.3% sand, 23.2% silt, 17.5% clay	71% sand, 17% silt, 13% clay	40% sand, 25% silt, 35% clay	40% sand, 25% silt, 35% clay	65.7% sand, 22.8% silt, 11.5% clay	67.5% sand, 21% silt, 2.8% clay	68% sand, 20% silt, 13% clay
**pH**	7.6	8.2	8.2	8.2	7.9	8.2	7
**Electrical Conductivity (dS/m)**	1	20	0.7	0.7	1	1	0
**World Reference Base (WRB) for Soil Resources/Food and Agriculture Organization of the United Nations (FAO) Soil Classifications**	Yl- Luvic Yermosols	Yl- Luvic Yermosols	Yl- Luvic Yermosols	Yl- Luvic Yermosols	Yl- Luvic Yermosols	Yl- Luvic Yermosols	Yl- Luvic Yermosols
**Number of Trees**	159	157	156	–	158	157	157

1. Climate data represents the annual mean for each location, over 30 years, from 4 km surrounding the coordinates, from 1991 to 2020, based on data from the PRISM Climate Group (2023; https://prism.oregonstate.edu/explorer/bulk.php).

2. Soil data is from United States Department of Agriculture (USDA), Natural Resources Conservation Service (2020).

3. Climate abbreviations are as follows: mean annual temperature (MAT) vapor pressure deficit (VPD). MAT Transfer is the MAT of the planting site minus the MAT of the source population.

4. Ecotype/adaptive trait syndrome is as described in Blasini et al. (2021; 2022).

5. WRB and FAO soil classifications are as described in Food and Agriculture Organizaiton of the United Nations (1992).

Tamarisk was removed from the tamarisk-invaded areas of the site with bulldozers during the spring and summer of 2017. Planting holes were dug 150 cm deep to plant cottonwoods as close to the water table as possible and preclude the need for irrigation. Trees were planted during November and December 2017. Replicates of trees stratified across ecotype and provenance were planted in random order within blocks and replicated in five blocks for each treatment. Trees were all approximately 150 cm tall (plus 76 cm of roots and potting soil) at the time of planting and were planted approximately 150 cm deep. Paired inoculated and uninoculated blocks within the tamarisk and non-tamarisk areas were separated by a minimum of 10 m to maintain inoculation treatment integrity over the two-year experiment. Cottonwood trees were planted with (inoculated treatments) or without (not inoculated treatments) 120 ml of live soil inoculum. Live soil inoculum was added to the field soil during planting by mixing it with field soil when filling in planting holes, in the uppermost 20 cm of the hole containing fine roots. In conjunction with the deep planting, trees were watered when planted but received little water after planting aside from natural precipitation. In addition to the arid climate of the region, and lack of irrigation provided to the trees in the experiment, the study took place during the worst drought since 800 CE in the southwestern United States (Williams et al., 2022).

**Figure 1:**
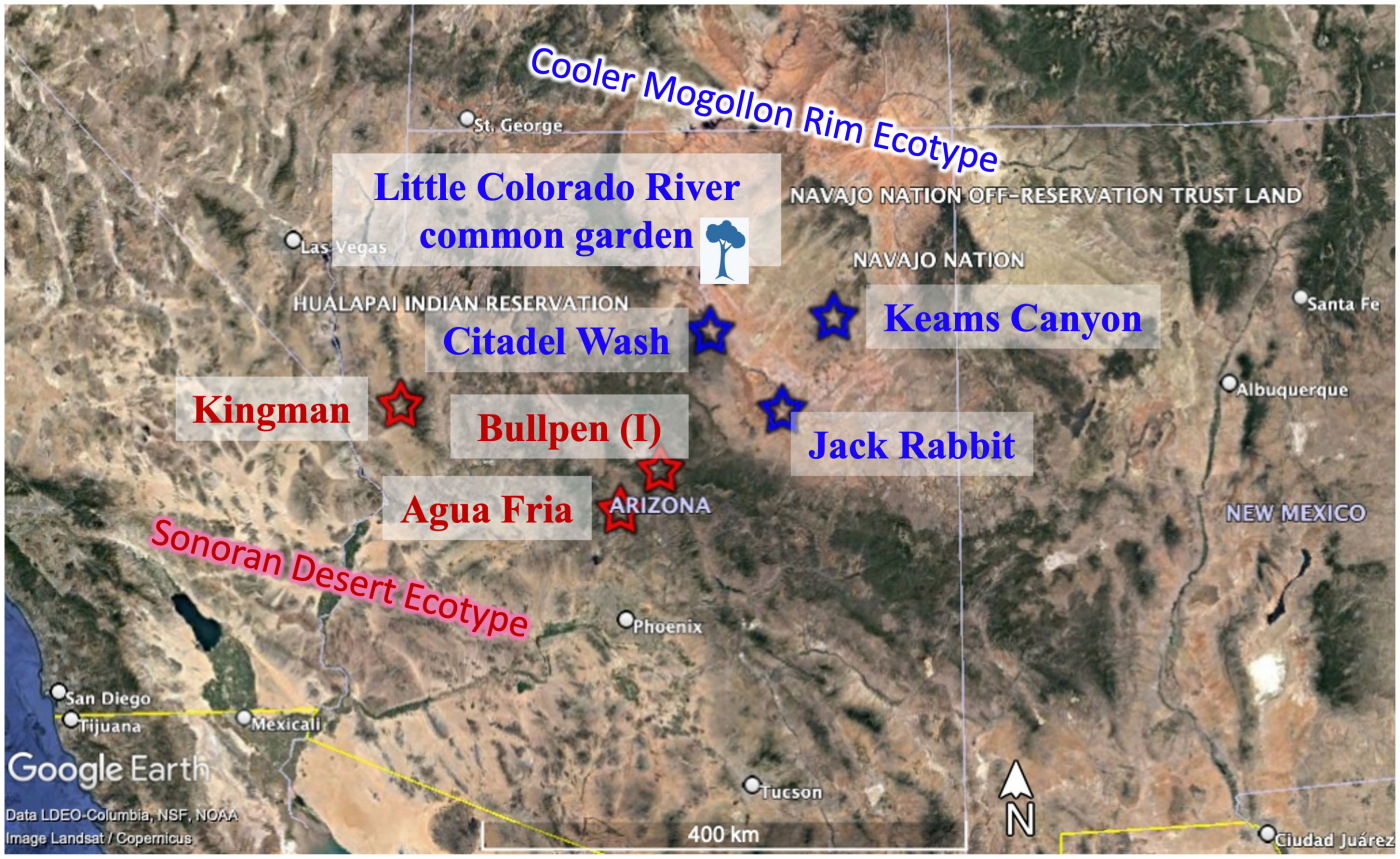
Map of cooler, Mogollon Rim (MR; blue) and warmer, Sonoran Desert (SD; red) ecotype source locations and Little Colorado River common garden (in the cooler, MR ecotype). Inoculum source is indicated with (I).

Locations of the common garden, and inoculum and tree sources are shown in **Figure 1**. Climate and soil data for the common garden and all source populations are presented in **Table 1**. The experimental design with the number of trees in each treatment can be found in **Supplementary Table 1**.

To create the live soil inoculum, living fine root fragments and rhizosphere soil were collected from Fremont cottonwoods in a healthy, tamarisk-free, Fremont cottonwood population from the SD ecotype (Bullpen; **Table 1**). This live soil was homogenized and then divided amongst ten 30 cm x 30cm x 30 cm plastic containers, mixed with 30% sterile clay particles. Plants known to associate with arbuscular and ectomycorrhizal fungi were cultivated in these containers in the greenhouse for ten months. Each container of the live soil was planted with arbuscular mycorrhizal hosts (leek [*Allium* sp.], corn [*Zea mays*], and marigolds [*Calendula* sp.]) and ectomycorrhizal hosts (ponderosa pines [*Pinus ponderosa*]) and Fremont cottonwoods. Cottonwoods came from the same ecotypes and populations that were used in the field experiment (which are known to associate with both arbuscular and ectomycorrhizal fungi under these conditions, e.g. Meinhardt and Gehring, 2012). The greenhouse experienced natural light and dark cycles, and the live soil inoculum plantings were watered twice per week. Plants in the live soil inoculum were cut at the base prior to inoculum use. Roots of the plants used to cultivate mycorrhizal fungi within the inoculum during greenhouse cultivation were left in the soil, and the soil was mixed to increase homogeneity of the contents and break-up roots into fragments.

### 2.3 Survival measurements to address hypotheses in the field experiment

Survival of each tree was measured in the field during July 2018 (year 1) and September 2019 (year 2). Survival was primarily evaluated by examining whether trees had green leaves during the growing season. If trees lacked green leaves during the growing season, tissues at the base of the stem under the surface examined to identify if they were still green and living or dried and dead. In a small area at the base of the stem, the thin surface layer was scraped away with a fingernail to reveal the nature of the tissues underneath.

As a result of tree size, planting and rooting depths, and project constraints that included restoration as a goal, collection of the fine root fragments necessary to confirm mycorrhizal status across treatments was not possible.

### 2.4 Statistical analyses for survival data from the field experiment

All statistical analyses were conducted in R version 4.0.3 (R Core Team, 2020). Individual trees were used as the independent experimental unit since plants were randomly planted and environmental heterogeneity was relatively low (as in Grady et al., 2011, 2015).

To analyze binomial survival data by treatment variables in conjunction with the random effects of tree source population, we utilized a generalized linear mixed effects model using the glmer() function in the lme4 package (Bates et al., 2015) in R. To establish an overall model for use, a suite of mixed effects models were evaluated. All mixed effects models included year one field survival and year two field survival as the dependent variables, and plant ecotype, and tamarisk and inoculation treatments as the independent variables. Mixed-effects models were evaluated using AIC scores to test whether the random variables (which were not the focus of the experiment) of plant source population and field plot replicate meaningfully improved the model without jeopardizing its statistical power (indicated by the additional warnings noted in **Supplementary Table 2**). The model using source population as a random effect was selected based on AIC scores for both year 1 and year 2 survival and the ability of the model to perform analyses for both years without additional warnings (**Supplementary Table 2**). Eight treatments (2 tamarisk x 2 inoculation x 2 ecotype) yielded over fifty specific contrasts that could have been evaluated with post-hoc tests using the emmeans package. However, the objective of the experiment was to evaluate the specific contrasts addressing our hypotheses: 1) the soil legacy left by prior tamarisk invasion would generally reduce plant survival after tamarisk removal; 2) live microbial soil/mycorrhizal restoration would improve the survival of trees in areas with a tamarisk soil legacy and 3) live microbial soil/mycorrhizal restoration would improve the survival of trees in areas without a tamarisk history; 4) assisted migrant trees from the warmer SD ecotype would have survival rates equal to or lower than those for trees from the local MR ecotype. As a result, individual contrasts that addressed the specific hypotheses of interest were evaluated within the overall model, using vectors to specify the four contrasts desired (Muldoon, 2019) in conjunction with the emmeans() function in the emmeans package. The sidak adjustment for multiple comparisons was used to adjust post-hoc results for the number of post-hoc comparisons made (Length, 2020). Hypothesis test results are presented as the emmeans z ratio (z, which can be generally described as the contrast divided by its standard error), resulting p-value (p).

Measures of effect size were obtained utilizing the odds ratios from the emmeans() function in the emmeans package (Length, 2020), specifying type=‘response’ to obtain results that were back-transformed from the logit scale (and can be seen in **Supplementary Table 6**). Probabilities of surviving and standard errors for graphical depictions are from these model results.

### 2.5 Greenhouse methods to assess inoculation efficacy

During May through August 2020, we conducted a greenhouse experiment that replicated the field study as closely as possible to confirm the efficacy of mycorrhizal inoculation utilizing the same methods and soils. In the greenhouse experiment, 80 small (~25 cm in length and 7 mm in diameter) Fremont cottonwoods (40 cuttings from the SD ecotype and 40 cuttings from the MR ecotype) were planted in D40 pots. Cottonwoods were planted in a mix of one-half potting soil and one-half live soil from the common garden. Half of the trees were planted in common garden soil from areas with a tamarisk legacy, and half in common garden soil from areas with no tamarisk legacy. The soil inoculum was added to cottonwoods during re-potting with common garden soil/potting soil mix. One half of the cottonwoods from each ecotype in each potting mix treatment received live, and one-half received sterilized, soil inoculum. Cottonwoods in each treatment were stratified across the same ecotypes as in the field experiment. Soil inoculum was sourced from a healthy, tamarisk-free, cottonwood population from the SD ecotype (Agua Fria; **Table 1**) and maintained in the greenhouse as described in Section 2.3 prior to being used for this experiment. Cottonwoods that died early in the experiment were replanted to maintain a balanced experimental design.

To confirm the efficacy of the inoculation methods used in the field, fine roots from the cottonwoods were collected 125 days after initiating treatments. Roots were cleaned and rinsed to remove soil, placed into plastic bags, and immediately frozen until evaluation. EMF have been shown to be more sensitive to tamarisk presence and assisted migration (Meinhardt and Gehring, 2012; Markovchick et al., 2023), but we measured both EMF and AMF colonization. Colonization was evaluated for trees in all treatments. Root samples were divided into subsamples for separate evaluation of EMF and AMF colonization. Root tips (living, dead [as evidenced by their shriveled, dried, and darkened nature], and living colonized by an EMF morphotype) were counted under a dissecting microscope using the gridline intersect method (as described in Brundrett et al., 1996). To evaluate EMF colonization across treatments, 7500 gridline intersections were evaluated across 75 trees, and 258 EMF root tips were identified and counted. Separate root subsamples were cleared and stained, and evaluated for AMF hyphae, vesicles, and arbuscules using the gridline intersection method under a compound light microscope for a subsample of 14 trees using the methods of Brundrett et al. (1996). Care was taken to quantify dark septate endophytes (DSE; non-mycorrhizal root fungi that have no specialized exchange structure) separately, as indicated by melanized septate hyphae.

### 2.6 Statistical analyses for the greenhouse inoculation efficacy test

All statistical analyses were conducted in R version 4.0.3 (R Core Team, 2020). Individual trees were used as the independent experimental unit since plants were randomly planted and environmental heterogeneity was relatively low (as in Grady et al., 2011, 2015).

To investigate the efficacy of inoculation, EMF colonization and AMF colonization from the greenhouse inoculation experiment were assessed using mixed effects linear regression with the lmer() function in the lme4 package (Bates et al., 2015) in R. Inoculation and tamarisk treatment was used as the predictor (independent) variable, population was used as a random variable, and EMF and AMF colonization were dependent variables.

## 3 Results

### 3.1 Overall model results from the field experiment

Independent variables (tamarisk, inoculation and ecotype treatments) demonstrated significant effects on the dependent variable (survival) in the overall model. During year one, source ecotype and tamarisk soil legacy treatment were significant main effects (Z=1.97 and p = 0.049; Z = −5.12, p < 0.001), with significant interactions for inoculation by tamarisk, source by tamarisk, and inoculation by source by tamarisk (Z = 1.99, p = 0.47; Z = −2.41, p < 0.05; Z = 2.46, p < 0.05). During year two, the inoculation treatment was the significant main effect (Z = −2.66, p < 0.01), with significant interactions for inoculation by source, and inoculation by tamarisk (Z = 2.206, p < 0.05; Z = 2.689, p < 0.01). Overall model results can be found in **Supplementary Table 4**. Model results for each treatment in each year can be found in **Supplementary Table 5**. Specific post-hoc contrasts for each hypothesis also yielded significant results, are discussed individually below, and can be found in **Supplementary Table 6**.

### 3.2 H1: tamarisk soil legacy negatively impacts cottonwood survival in the field

**Figure 2:**
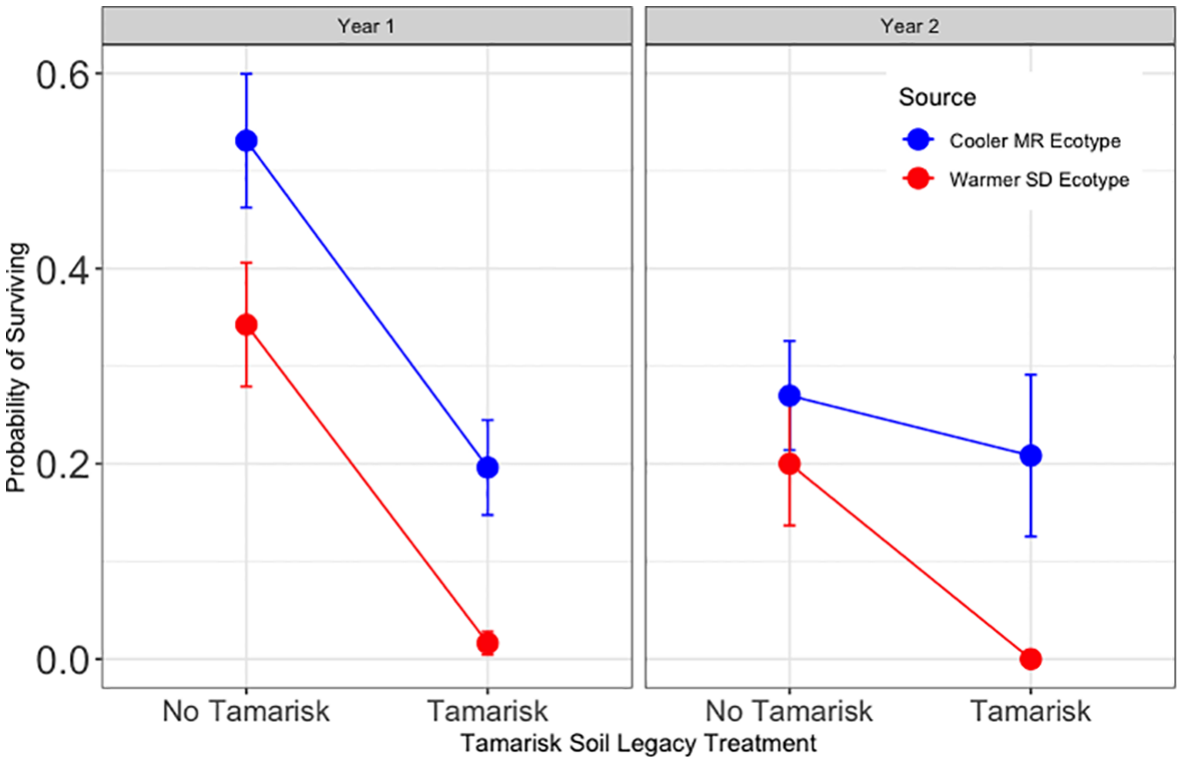
Results for Hypothesis 1. Probability of surviving for trees without inoculation sourced from the cooler, local Mogollon Rim (MR; blue) ecotype and from the warmer Sonoran Desert (SD; red) ecotype in each tamarisk legacy treatment. Points represent model means and error bars represent 1 SE. Across ecotypes and source, first year survival for trees in tamarisk soil was 8.3% of that for trees in soil with no tamarisk legacy (Z = −6.26, p<0.001).

Hypothesis 1 was supported for year one (**Figure 2**). Without inoculation treatments, trees planted in tamarisk soil were significantly less likely to survive year one than trees planted in soil without a tamarisk legacy (z = −6.26, p <0.001). Survival of trees in tamarisk soil was 8.3% of that for trees in soil without a tamarisk legacy. Year 2 results were not significant (**Supplementary Table 6**).

### 3.3 H2: inoculation with live soil increases field survival of trees in tamarisk legacy soil

**Figure 3:**
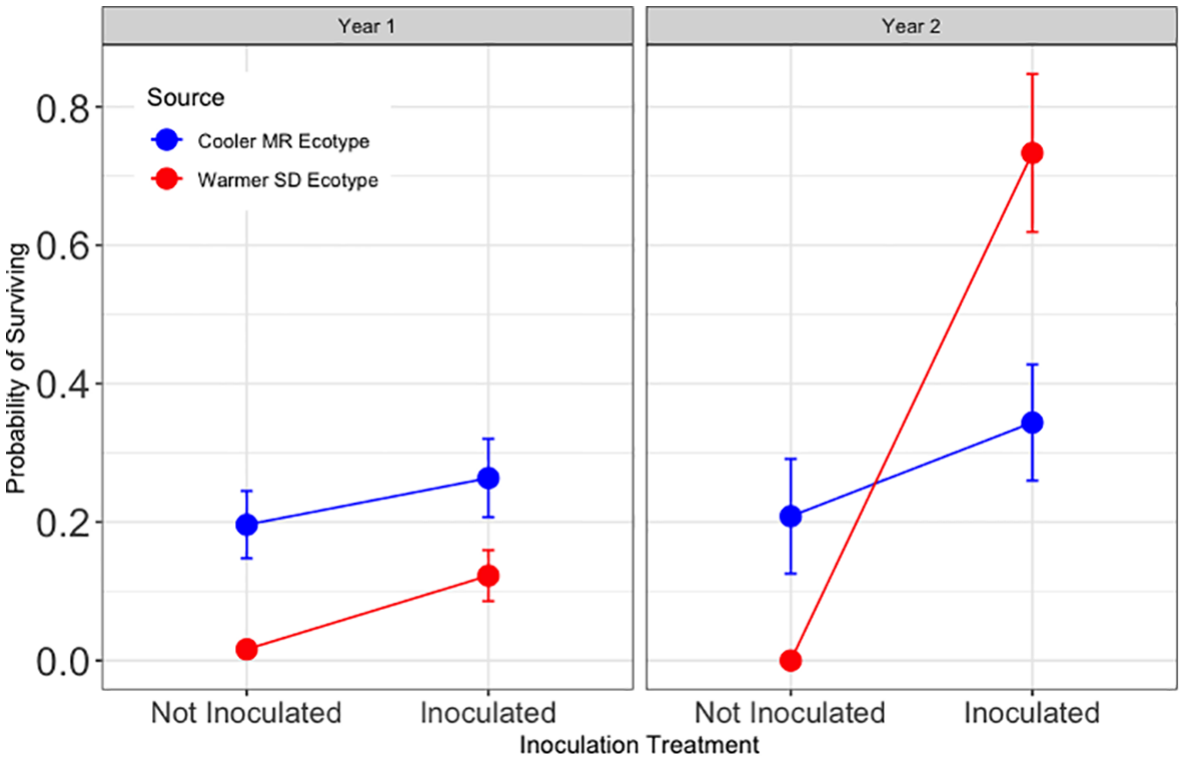
Results for Hypothesis 2. First and second year survival (left and right, respectively) for trees in tamarisk soil legacy from the cooler, local Mogollon Rim (MR; blue) ecotype and from the warmer Sonoran Desert (SD; red) ecotype. Points represent model means and error bars represent 1 SE. Inoculation more than tripled year one survival in tamarisk soil (Z = -3.05, and p < 0.01)). The significant effects of inoculation interacted with source and tamarisk soil legacy treatment during year two (Z = −2.66, p<0.01; Z = 2.21, p<0.05; Z = 2.69, p <0.01 respectively).

Hypothesis 2 was partially supported (**Figure 3**). For trees in tamarisk soil, year one survival was significantly higher with inoculation than without (z = −3.05, and p < 0.01). Survival in tamarisk soil if inoculated was 350% of that for trees that were not inoculated. During year two, inoculation results in tamarisk soil depended on tree source ecotype, as visualized in **Figure 3**, and demonstrated by the significant main effect of inoculation and significant interactions with source ecotype and tamarisk legacy soil in year two seen in the model results (Z = −2.66, p<0.01; Z = 2.21, p<0.05; Z = 2.69, p <0.01 respectively).

### 3.4 H3: inoculation with live soil increases field survival of trees in soil without a tamarisk legacy

**Figure 4:**
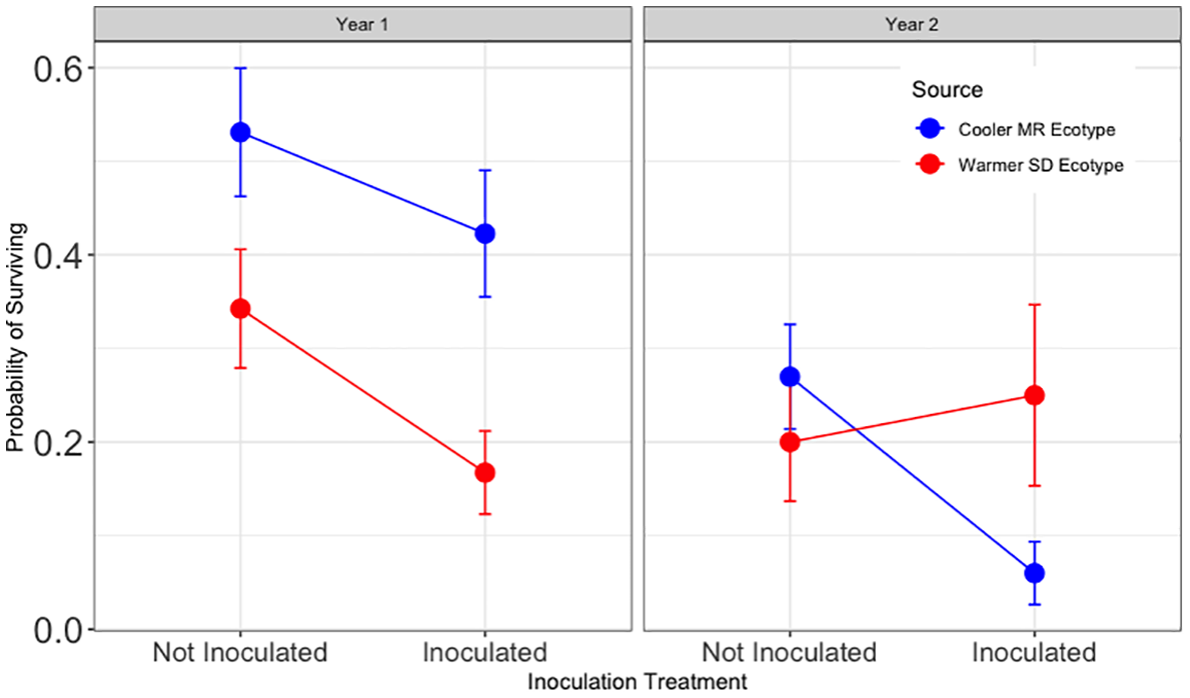
Results for Hypothesis 3. First and second year survival for trees in the common garden blocks without a tamarisk legacy. Shades of blue show results for trees sourced from the cooler Mogollon Rim (MR) local ecotype and shades of red show results for assisted trees sourced from the warmer Sonoran Desert (SD) ecotype. Points represent model means and error bars represent 1 SE. In soil without a tamarisk legacy, inoculated trees had one half the survival of non-inoculated trees (p<0.01). During the second year, an interaction between inoculation and tree ecotype (Z = 2.21, p < 0.05) resulted in no significant effect for inoculation overall.

Hypothesis 3 was not supported (**Figure 4**). For trees in soil without a tamarisk legacy, year one survival was significantly lower with live soil inoculum than without (z = −3.36, and p <0.01). Year one survival in soil without a tamarisk legacy if provided live inoculum was 50% of that for trees that did not receive inoculum.

For trees in soil without a tamarisk legacy, year two survival was not significantly different overall for cottonwoods that received inoculum and those that did not. (**Supplementary Table 6**). As can be seen in **Figure 4**, the overall model results reveal a year two interaction between the main effect of inoculation (z = −2.66, p < 0.01) and tree source ecotype (z = 2.21, p < 0.05).

### 3.5 H4: with inoculation using live soil from the same ecotype, assisted migrants will have survival rates in the field lower than or equal to those for trees from the local ecotype

**Figure 5:**
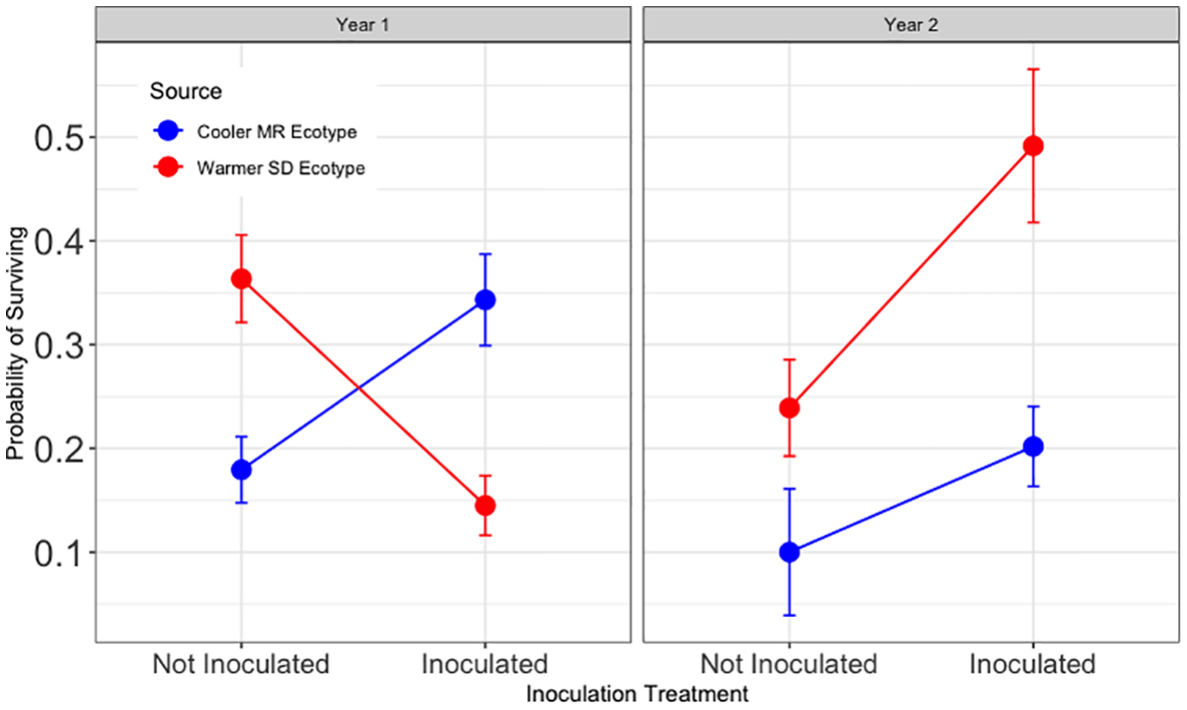
Results for Hypothesis 4. First and second year survival for trees in the common garden sourced from the cooler, local Mogollon Rim (MR; blue) ecotype and from the warmer Sonoran Desert (SD; red) ecotype in each inoculation treatment (combined data from **Figures 4**, **5**). Points represent model means and error bars represent 1 SE. Inoculated assisted migrant trees from the warmer ecotype (SD) had a lower chance of survival during year one (Z = −3.03, p = 0.010) and a higher chance of surviving during the second year than trees from the cooler, local ecotype (MR) across tamarisk soil treatments (Z = 3.16, and p < 0.006).

Hypothesis 4 was supported during year one and not supported during year two (**Figure 5**). During year one, inoculated trees from the warmer (SD) ecotype had significantly lower survival across tamarisk soil legacy treatments (z = −3.03, p < 0.05). During year two, survival for inoculated assisted migrant trees was significantly higher for than for those from the local ecotype across tamarisk soil legacy status (z = 3.16, and p < 0.01). Survival of inoculated trees during year two for assisted migrants was 520% of that for trees from the local ecotype.

### 3.6 Greenhouse test of inoculation efficacy

**Figure 6:**
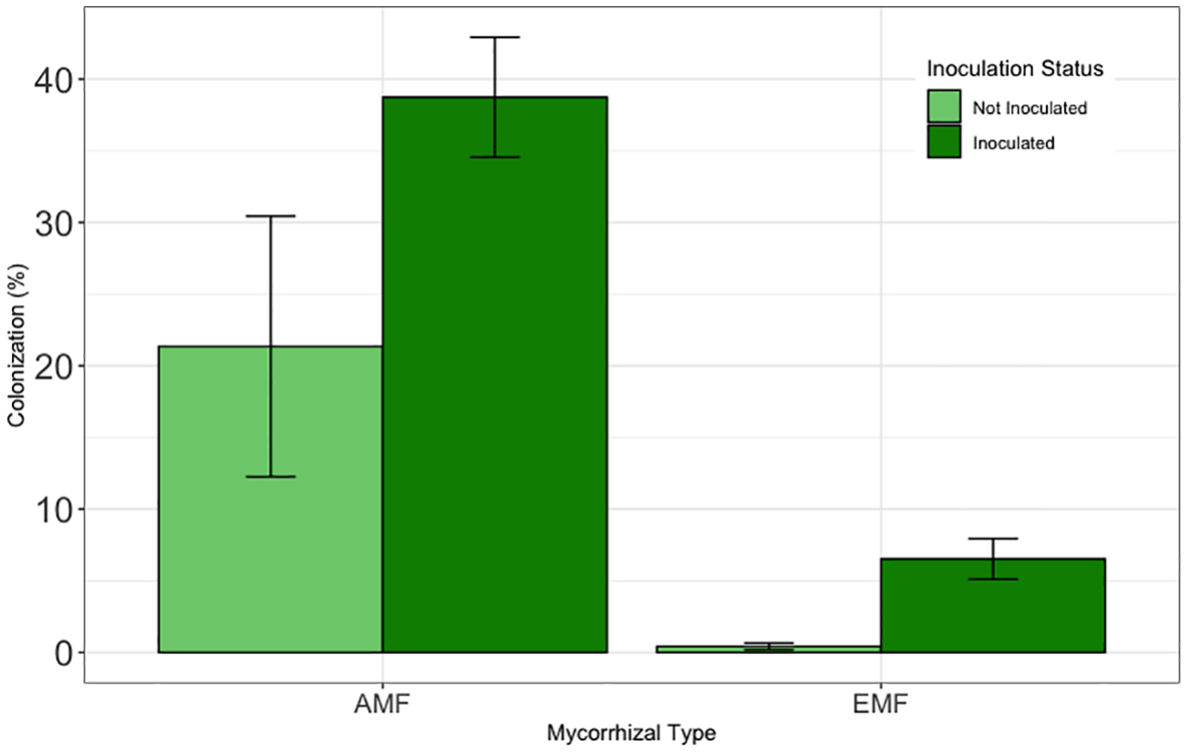
Results from greenhouse inoculation efficacy test. To be comparable to the field experiment, field and potting soil were not sterilized prior to inoculation test. Bars are means and error bars represent 1 SE. Inoculation increased EMF colonization by nearly 500% (p<0.001) and AMF colonization by nearly 100% (p<0.05).

Inoculation significantly increased both EMF (F = 16.26, df = 1, 71, and p < 0.001) and AMF (F=10.20, df=1, 9.68, p <0.01) colonization (**Figure 6**). Averaged across tamarisk treatments and tree source, inoculation increased the number of EMF root tips by nearly 500% (df=67, t=4.06, p<0.001). Averaged across tamarisk treatments and tree source, inoculation nearly doubled AMF colonization (df=67, t=4.06, p<0.001). Means and standard errors can be found in **Supplementary Table 3**.

## 4 Discussion

Due to the variety of benefits associated with native mycorrhizal symbioses, reductions and shifts in their communities due to invasive species and other disturbances are concerning, particularly as the stressors on plant communities (that mycorrhizae are known to help mitigate) increase under climate change. For example, mycorrhizal fungi increase plant access to nutrients, mediate responses to stressors, pests, and climate (Babikova et al., 2013; Kivlin et al., 2013; Wilkinson and Dickinson, 1995), improve plant water use efficiency (Querejeta et al., 2003; 2006), and provide access to deep water (Bornyasz et al., 2005; Querejeta et al., 2007). Although more challenging to prove definitively and the focus of a current debate (Karst et al., 2023; Klein et al., 2023), some evidence suggests that certain mycorrhizae may also share water and other resources among plants (Egerton-Warburton et al., 2007; Teste and Simard, 2008; Klein et al., 2016). While few studies have examined whether the eradication of invasive species permits recovery of the mycorrhizal fungal communities and their services, limited data suggests recovery times could be extensive. While fungal biomass began to recover following three years of garlic mustard (*Alliaria petiolota*) removal in eastern deciduous forests, fungal community composition and soil physical and chemical properties remained similar to those in invaded sites (Anthony et al., 2019). Three years after eradication of the invasive plant, fungal community composition, and soil chemical and physical properties, remained comparable to invaded communities. Other studies similarly suggest that disruptions to mycorrhizal fungal communities have long-term effects, which natural dispersal and planting materials and processes do not successfully mitigate (Sykorova et al., 2007; Southworth et al., 2009; Peay et al., 2010; Pankova et al., 2018).

In this study, the soil legacy left by exotic tamarisk invasion reduced first year survival of native cottonwood trees by over 90%, even after tamarisk trees were removed from the area. In areas with this tamarisk soil legacy, inoculation with native mycorrhizal fungi from native, uninvaded riparian areas significantly improved first-year cottonwood survival across trees from both ecotypes, more than tripling the survival rate. In soils without a tamarisk legacy, the effect of inoculation was more variable, interacting tree source ecotype and time since planting. Although assisted migrant SD trees inoculated with native, living soil inoculum from their home ecotype had a more challenging first year, they survived the second year in extremely harsh conditions at over five times the rate of inoculated local MR trees, revealing the real restoration implications for appropriate and inappropriate soil biome/plant pairings.

### 4.1 Greenhouse inoculation efficacy

Inoculation with live soil from non-invaded cottonwood riparian areas significantly increased EMF and AMF colonization of cottonwoods compared to non-inoculated controls in the greenhouse. These results provide support for our field experiment methods where it was not possible to sample roots over the two year observation period. Although colonization rates were fairly low, relatively small increases in colonization have been associated with relatively large effects on plant survival in cottonwoods. For example, in another recent intraspecific assisted migration study with Fremont cottonwoods (Markovchick et al., 2023), slight increases in colonization (from 0% to 5%) were associated with 46% of the variation in survival for the tree population with the lowest survival rates.

### 4.2 The effects of invasive vegetation

Our study provides evidence that soil legacies left by an invasive exotic species such as tamarisk can impact native plants negatively after the removal of the invasive species itself. This is consistent with previous studies showing that invasive species and shift mycorrhizal communities, reduce plant survival and/or biomass, and alter mycorrhizal colonization (Carey et al., 2004; Meinhardt and Gehring, 2012; Wilson et al., 2012). Direct measures of mycorrhizal communities (Pankova et al., 2018; Anthony et al., 2019; Duell et al., 2023) and the success of restoring soil and mycorrhizal communities along with vegetation (Wubs et al., 2016; Koziol and Bever, 2017) provide evidence that the effects of disturbances on mycorrhizal communities can last for years. The negative effects on native plants and on mycorrhizal mutualisms from invasion by exotic plant species have now been documented across multiple systems. Further research is needed to determine the exact limitations and mechanisms operating, and how ecosystem services provided by mycorrhizal fungi and the soil microbiome, such as soil aggregation and water access (e.g. Egerton-Warburton et al., 2007; Duell et al., 2023), are impacted.

### 4.3 Implications for assisted migration

Two types of assisted migration are generally recognized (Pedlar et al., 2012). First, species rescue assisted migration to avoid extinction of species threatened by climate change, and second, forestry assisted migration to identify populations and genotypes that would achieve higher survival and growth with climate change than local stock, which is focused on widespread and commercially important species. Keith et al. (2023) identified a third type as community assisted migration, which is focused on the dependent or associated communities of plant species. This third type may be especially important with essential plant mutualists such as pollinators, dispersal agents and mycorrhizal fungi that affect plant performance. Such species have been referred to as the holobiome (usually a multicellular host and its microbial symbionts, as the primary unit of selection (e.g., Gilbert et al., 2012; Bordenstein and Theis, 2015). To the degree that this concept is correct, assisted migration that incorporates the holobiome should achieve higher success than assisted migration strategies that ignore these crucial interacting community members. Our findings showing that inclusion of mycorrhizal fungal mutualists results in much greater plant performance supports this concept.

Though it is challenging to address multiple stressors at once within field studies, it is nonetheless important to understand what results can be expected from many interacting factors. In this study, we have shown that small, incremental steps in assisted migration (Grady et al., 2015), even across ecotype or adaptive trait boundaries (Cooper et al., 2019; Blasini et al., 2021), can be similarly successful to local plant provenances under multiple stressors, if given appropriate mycorrhizal and live soil inoculum. This is consistent with results from Remke et al. (2020) suggesting that inoculation with microbiota from a plant provenance’s home environment, while most effective in home soil, can help ameliorate the negative impacts of growing at hotter, drier sites. The results of the current experiment indicate that increasing intraspecific genetic diversity through assisted migration (Etterson et al., 2020; Gomory et al., 2020; Saenz-Romero et al., 2021) with appropriate mycorrhizal inoculation in restoration is a promising strategy for climate change adaptation for some locations. However, more research is needed to determine the most appropriate or beneficial plant/inoculum pairings Allsup et al. (2023) found that inoculating plants with microbiota adapted to climatic characteristics of the planting site enhanced plant survival. In other (e.g. Johnson et al., 2010, 2014) and the current study, inoculum from the same location or (in the current study) ecotype as the plants appears most effective. This suggests that the current study makes an important contribution to our understanding of how to best pair plant microbial source materials for restoration, replanting, and assisted migration. The current study suggests that even within the same plant species, ecosystems, and region, microbial pairings may need to match plant ecotype or physiological adaptations. For example, cottonwoods with physiological adaptations to frost (the local, cooler, MR ecotype; Hultine et al., 2020; Blasini et al., 2021, 2022) did not seem to benefit from microbial inoculations adapted to warmer, SD ecotype conditions (where trees in the same species are adapted to heat; Hultine et al., 2020; Blasini et al., 2021, 2022) unless there was a history of tamarisk.

The long-term effects of assisted migration on interactions between plants and their microbiomes still need to be investigated. For example, what might the effects of assisted migration be on the ability of trees to form common mycorrhizal networks that exchange nutrients, water, and pest signals (e.g. Bingham and Simard, 2011; Babikova et al., 2013; Klein et al., 2016), and how that is affected by including plants and mycorrhizal fungi from multiple provenances, for example? Additionally, given the different physiological adaptations of the two ecotypes, how will differences in their physiological adaptations (such as leaf size, leaf duration, ability to move water for evaporative cooling, etc.), and potentially their microbiomes, alter the albedo of the landscape, the ability of the canopy to provide microclimate buffering, and the carbon cycling of the ecosystem (Michalet et al., 2023)?

This reinforces the merits of considering the interaction of the organism and its microbiome in replanting, restoration, and climate change adaptation (Zilber-Rosenberg and Rosenberg, 2008; Bordenstein and Theis, 2015; Whitham et al., 2020; Allsup et al., 2023), and the urgent need for large-scale assisted migration studies that address inter-species interactions (Bucharova, 2017).

Research on community genetics also indicates that the interactions seen here between native plant ecotypes, soil microbes, and invasive plants could likely have broader implications including for insects, lichens, pathogens, endophytes and other organisms (e.g. Lamit et al., 2015; Keith et al., 2023). In fact, one recent paper (Argüelles-Moyao and Galicia, 2023) suggested that the soil microbiome and fungal species should be primary considerations in assisted migration and the selection of sites for afforestation, since they are as important as temperature and precipitation to outcomes, and regulate key ecosystem processes.

### 4.4 The impact of time and timing

In addition to interactions with tree ecotype and tamarisk soil legacies, this study found differences in the impact of inoculation treatments between the first and second growing seasons after planting. We are aware of at least two studies demonstrating that the timing of inoculation can impact its initial effectiveness, because establishing the mycorrhizal symbiosis, particularly under otherwise stressful conditions, imposes an initial cost on the plant (Mortimer et al., 2005; Maltz and Treseder, 2015). However, the clear impacts of timing on field survival for trees in non-tamarisk soil in the current study (negative the first year, and interacting with ecotype during the second year) demonstrate the importance of this consideration in a manner that we have not seen in the literature to date. Given the extremely harsh conditions at the experimental site and absence of irrigation or other types of support for new plantings, results suggest that inoculation at stressful times such as field planting might best be accompanied by adequate watering and support for new plantings. Although complicated by multiple interactions, the positive trend for SD trees appropriately paired with soil inoculum during the second year even in non-tamarisk soil legacies suggest that the beneficial effects from appropriately paired inoculum might grow with time, similar to findings from Neuenkamp et al. (2019).

### 4.5 Interactions between site conditions, and provenances of native plants and mycorrhizal fungi

The widespread success of mycorrhizal restoration is remarkable (Neuenkamp et al., 2019), especially considering the continued prevalence of mass-produced mycorrhizal inoculums (Hart et al., 2017; Saloman et al., 2022). Given extensive evidence showing that mycorrhizal symbioses are impacted by and co-evolved with plant provenances and site conditions (Johnson et al., 1992, 2010, 2014; Rua et al., 2016), it is unsurprising that in non-tamarisk soil the effects of mycorrhizal inoculation depended on the pairings between the provenance of plants and the mycorrhizal fungi utilized. However, due to the presence of other invasive plant species and the lack of remaining cottonwood trees to provide fungal inoculum at the site, it seemed reasonable to hypothesize that inoculation with a diverse mix of native mycorrhizal fungi native to cottonwood trees in a riparian area in the same state would be broadly beneficial across plant provenances and ecotypes. Interestingly, even taking into consideration the effects of timing, our study did not support this. Further research is needed to reveal how pairing mycorrhizal inoculum sources and plant provenances can optimize results. For example, developing inoculum combinations that include microbiota from high salinity sites could be used to assist paired plant partners at a replanting site with high salinity, but it is not yet apparent whether plants of all intraspecies provenances would benefit equally, or what combination of mycorrhizal inoculation sources would best promote plant provenances of varying sources. As best practices for restoration begin to incorporate mycorrhizal restoration, there is an urgent need for additional research on mismatched verses optimal inoculum-plant pairings to avoid unintended, counterproductive negative impacts from a tool that has so much potential. Additionally, the physiological strategies of plants can vary by ecotype and population (e.g. Blasini et al., 2021; 2022). The outcomes of mycorrhizal symbiosis are known to vary with every change in partner and environment (Klironomos, 2003; Rillig and Mummey, 2006), and specific, limited subsets of mycorrhizal fungi within a community are known to provide certain services (e.g. Egerton-Warburton et al., 2007). Sevanto et al. (2023) recently demonstrated that within the same plant community, the outcomes of a pairing with a single fungal partner can vary based on tree genotype. These findings point to the importance of utilizing live, native soil inoculum, and of refining our understanding of optimal pairings between site conditions, plant source, and fungal inoculum source based on how plant physiological adaptations and fungal services co-vary. In some places, collection of live soil inoculum may not be possible (due to legal, archeological, and/or pathogenic concerns, for example), and/or scaling activities up to produce the needed volumes of live soil inoculum necessary may prove prohibitive. In our study, we collected relatively insignificant volumes of live soil inoculum (approximately two five gallon buckets or 38 liters) and propagated it in the greenhouse using methods to encourage growth of the mycorrhizal community. Such methods may reduce barriers to using live soil inoculum (making the practice more sustainable without requiring large volumes of living soil from natural areas), but could also potentially bias the resulting mycorrhizal community such that its overall makeup differs from exactly what it would be, or what might be optimal for the plants, in the field (e.g. Sykorova et al., 2007; Southworth et al., 2009).

### 4.6 Conclusions

This study demonstrated a large legacy effect of an invasive species on habitat restoration, and that field survival of assisted migrant plant provenances can be boosted by implementing intentional assisted migration of their soil biota and mycorrhizal fungi. This study provided evidence that appropriate, native soil inoculation could increase the efficacy of assisted migration from warmer areas as a climate change adaptation strategy, and that native soil inoculum should be sourced from the same ecotype as the plants being inoculated. These findings improve our understanding of fundamental ecological concepts about how invasive species and symbioses affect ecosystems and provide restoration best practice targets.

## Data availability statement

The raw data supporting the conclusions of this article will be made available by the authors, without undue reservation.

## Author contributions

LM: Conceptualization, Data curation, Formal analysis, Investigation, Methodology, Supervision, Visualization, Writing – original draft, Writing – review & editing. AB-A: Investigation, Writing – review & editing. DR: Data curation, Investigation, Writing – review & editing. TD: Data curation, Investigation, Writing – review & editing. KG: Conceptualization, Investigation, Resources, Writing – review & editing. KH: Methodology, Resources, Writing – review & editing. GA: Conceptualization, Resources, Writing – review & editing. TW: Conceptualization, Funding acquisition, Project administration, Resources, Supervision, Writing – review & editing. JQ: Conceptualization, Formal analysis, Investigation, Methodology, Resources, Supervision, Writing – review & editing. CG: Conceptualization, Funding acquisition, Investigation, Methodology, Project administration, Resources, Supervision, Visualization, Writing – review & editing.
