# Supplementary material for: Utilizing symbiotic relationships and assisted migration in restoration to cope with multiple stressors, and the legacy of invasive species
Source: Front Microbiomes. 2024 Mar 26;3:1331341. doi: 10.3389/frmbi.2024.1331341 (PMC12993495; doi:10.3389/frmbi.2024.1331341)
Supplement: Supplementary file 1 [file Table_1.docx]

Supplementary Material

# Supplementary Figures and Tables

## Supplementary Tables

**Supplementary Table 1.** Experimental design with number of trees in each treatment.

| **Tamarisk Soil Legacy** | **Inoculation Treatment** | **Ecotype Source** | **n** |
| --- | --- | --- | --- |
| No Tamarisk | Not Inoculated | Cooler MR Ecotype | 119 |
| No Tamarisk | Not Inoculated | Warmer SD Ecotype | 116 |
| No Tamarisk | Inoculated | Cooler MR Ecotype | 118 |
| No Tamarisk | Inoculated | Warmer SD Ecotype | 117 |
| Tamarisk | Not Inoculated | Cooler MR Ecotype | 117 |
| Tamarisk | Not Inoculated | Warmer SD Ecotype | 119 |
| Tamarisk | Inoculated | Cooler MR Ecotype | 118 |
| Tamarisk | Inoculated | Warmer SD Ecotype | 120 |

**Supplementary Table 2.** Overall mixed effects models considered. The model selected based on AIC values for both years and ability to model results without additional warnings appears in bold.

| **Response Variable** | **Year 1 Survival (n=944)** | | **Year 2 Survival (n=246)** | |
| --- | --- | --- | --- | --- |
| **Model** | **AIC** | **Warnings** | **AIC** | **Warnings** |
| **Survival ~ Tamarisk * Inoculation * Ecotype + (1\|Pop)** | **955** | **-** | **259** | **-** |
| Survival ~ Tamarisk * Inoculation * Ecotype + (1\|Pop) + (1\|Plot) | 952 | **-** | 236 | Yes |
| Survival ~ Tamarisk * Inoculation * Ecotype + (1\|Plot) | 964 | **-** | 234 | **-** |

**Supplementary Table 3.** EMF and AMF colonization sample sizes, means and standard errors from the greenhouse inoculation efficacy test.

| **Inoculation Treatment** | **EMF Sample** | | | **AMF Subsample** | | |
| --- | --- | --- | --- | --- | --- | --- |
|  | **N** | **Mean** | **SE** | **N** | **Mean** | **SE** |
| Not Inoculated | 38 | 0.42 | 0.23 | 6 | 21.35 | 9.09 |
| Inoculated | 37 | 6.53 | 1.42 | 8 | 38.74 | 4.19 |

**Supplementary Table 4.** Model main effects and interactions for each year.

| **Year** | **Effect** | **Z** | **p** |
| --- | --- | --- | --- |
| Year 1 | Inoculation Treatment | -1.63 | 0.103 |
| Year 1 | **Source Ecotype** | **-1.97** | **0.049*** |
| Year 1 | **Tamarisk Treatment** | **-5.12** | **0.000***** |
| Year 1 | Inoculation x Source | -1.25 | 0.211 |
| Year 1 | **Inoculation x Tamarisk** | **1.99** | **0.047*** |
| Year 1 | **Source x Tamarisk** | **-2.41** | **0.016*** |
| Year 1 | **Inoculation x Source x Tamarisk** | **2.46** | **0.014*** |
| Year 2 | **Inoculation Treatment** | **-2.66** | **0.008**** |
| Year 2 | Source Ecotype | -0.81 | 0.422 |
| Year 2 | Tamarisk Treatment | -0.59 | 0.556 |
| Year 2 | **Inoculation x Source** | **2.21** | **0.027*** |
| Year 2 | **Inoculation x Tamarisk** | **2.69** | **0.007**** |
| Year 2 | Source x Tamarisk | -0.09 | 0.930 |
| Year 2 | Inoculation x Source x Tamarisk | 186.96 | 0.930 |

*** p<0.05**

****p<0.01**

*****p<0.005**

**Supplementary Table 5.** The number of trees, and model results for the probability of surviving, in each treatment in each year.

| **Year** | **Tamarisk Soil Legacy Treatment** | **Source** | **Inoculation Treatment** | **Probability of Surviving** | **SE** | **df** | **n** |
| --- | --- | --- | --- | --- | --- | --- | --- |
| Year 1 | No Tamarisk | Cooler MR Ecotype | Inoculated | 0.423 | 0.07 | Infinite | 118 |
| Year 1 | No Tamarisk | Cooler MR Ecotype | Not Inoculated | 0.531 | 0.07 | Infinite | 119 |
| Year 1 | No Tamarisk | Warmer SD Ecotype | Inoculated | 0.167 | 0.04 | Infinite | 117 |
| Year 1 | No Tamarisk | Warmer SD Ecotype | Not Inoculated | 0.343 | 0.06 | Infinite | 116 |
| Year 1 | Tamarisk | Cooler MR Ecotype | Inoculated | 0.264 | 0.06 | Infinite | 118 |
| Year 1 | Tamarisk | Cooler MR Ecotype | Not Inoculated | 0.196 | 0.05 | Infinite | 117 |
| Year 1 | Tamarisk | Warmer SD Ecotype | Inoculated | 0.123 | 0.04 | Infinite | 120 |
| Year 1 | Tamarisk | Warmer SD Ecotype | Not Inoculated | 0.016 | 0.01 | Infinite | 119 |
| Year 2 | No Tamarisk | Cooler MR Ecotype | Inoculated | 0.060 | 0.03 | Infinite | 50 |
| Year 2 | No Tamarisk | Cooler MR Ecotype | Not Inoculated | 0.270 | 0.06 | Infinite | 63 |
| Year 2 | No Tamarisk | Warmer SD Ecotype | Inoculated | 0.250 | 0.10 | Infinite | 20 |
| Year 2 | No Tamarisk | Warmer SD Ecotype | Not Inoculated | 0.200 | 0.06 | Infinite | 40 |
| Year 2 | Tamarisk | Cooler MR Ecotype | Inoculated | 0.344 | 0.08 | Infinite | 32 |
| Year 2 | Tamarisk | Cooler MR Ecotype | Not Inoculated | 0.208 | 0.08 | Infinite | 24 |
| Year 2 | Tamarisk | Warmer SD Ecotype | Inoculated | 0.733 | 0.11 | Infinite | 15 |

**Supplementary Table 6.** Contrast results for each hypothesis in each year.

| **Year** | **Contrast** | **Odds Ratio** | **SE** | **df** | **z** | **p** |
| --- | --- | --- | --- | --- | --- | --- |
| Year 1 | **H1** | **0.083** | **0.03** | Inifinite | **-6.26** | **0.000***** |
| Year 1 | **H2** | **3.521** | **1.45** | Inifinite | **3.05** | **0.009**** |
| Year 1 | **H3** | **0.499** | **0.10** | Inifinite | **-3.36** | **0.003***** |
| Year 1 | **H4** | **0.327** | **0.12** | Inifinite | **-3.03** | **0.010*** |
| Year 2 | H1 | 2.3E-06 | 0.60 | Infinite | -5E-05 | 1.000 |
| Year 2 | H2 | 1.7E+06 | 437758005814.46 | Infinite | 5.58E-05 | 1.000 |
| Year 2 | H3 | 4.8E-01 | 0.22 | Infinite | -1.59 | 0.381 |
| Year 2 | **H4** | **5.2** | **2.75** | Infinite | **3.16** | **0.006**** |

*** p<0.05**

****p<0.01**

*****p<0.005**
